# Balancing diagnostic accuracy and cost in the follow‐up of histological low‐grade squamous intraepithelial lesions

**DOI:** 10.1111/aogs.70255

**Published:** 2026-05-22

**Authors:** Riina Aarnio, Vasudha Ahuja, Linda Ryen, Lovisa Bergengren

**Affiliations:** ^1^ Department of Women's and Children's Health, Uppsala University, Uppsala, Sweden, Department of Obstetrics and Gynaecology Danderyd University Hospital Stockholm Sweden; ^2^ Clinical Epidemiology and Biostatistics, School of Medical Sciences, Faculty of Medicine and Health Örebro University Örebro Sweden; ^3^ Faculty of Medicine and Health, University Health Care Research Center Örebro University Örebro Sweden; ^4^ Department of Women's Health, Faculty of Medicine and Health Örebro University Örebro Sweden

**Keywords:** colposcopy, cost‐effectiveness analysis, follow‐up studies, human papillomavirus, low‐grade squamous intraepithelial lesion

## Abstract

**Introduction:**

Primary cervical screening with testing for human papillomavirus (HPV) brings increased detection of women with low‐grade squamous intraepithelial lesion recommended for follow‐up with colposcopy. This study evaluates whether co‐testing (HPV + cytology) or testing for HPV alone can safely replace colposcopy as follow‐up for histologically confirmed low‐grade squamous intraepithelial lesions. It estimates these strategies' diagnostic performance in detecting histological high‐grade squamous intraepithelial or more severe lesions at follow‐up and analyzes their relative cost‐effectiveness.

**Material and Methods:**

This is a retrospective cohort study on follow‐up data of women aged 23–70 with histological low‐grade squamous intraepithelial lesion, diagnosed during 2017–2018, with an analysis of data from the Swedish National Cervical Screening Registry. Out of the 14 643 women with low‐grade squamous intraepithelial lesions, 4213 women with complete data on HPV, cytology, and histology results were analyzed. The primary outcome was to estimate the diagnostic performance of the strategies of co‐testing (HPV + cytology) and human papillomavirus‐alone in detecting histological high‐grade squamous intraepithelial or more severe lesions in the follow‐up of histological low‐grade squamous intraepithelial lesions. The secondary outcome was to perform a cost‐effectiveness analysis of these strategies.

**Results:**

Using co‐testing as the primary follow‐up and reserving colposcopy for women who tested positive for HPV or cytology results yielded a sensitivity of 96% for high‐grade squamous intraepithelial or more severe lesions. HPV‐alone testing had 88% sensitivity. Adding colposcopy to co‐testing would require 1735 (70%) additional examinations to detect 14 (3.9%) more women with high‐grade squamous intraepithelial or more severe lesions. No cancer cases were missed by co‐testing or HPV‐alone. The cost of detecting one extra high‐grade squamous intraepithelial or more severe lesion by adding colposcopy to co‐testing was 81 959 EUR.

**Conclusions:**

Histological low‐grade squamous intraepithelial lesion follow‐up can be safely managed with co‐testing, considerably reducing the number of colposcopies and associated costs.

## Graphical abstract

Primary follow‐up of histological LSIL can shift from colposcopy to liquid‐based co‐testing without missed cancers, as the incremental cost per additional HSIL+ detected is unjustified.

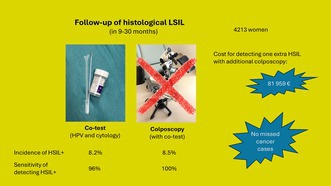

**Abbreviations**
AIS: adenocarcinoma in situ
AUC: area under the curve
CI: confidence intervals
CIN: cervical intraepithelial neoplasia
EUR: Euros
FN: false negative
FP: false positive
HPV: human papillomavirus
HSIL: high‐grade intraepithelial lesion
HSIL+: high‐grade squamous intraepithelial or more severe lesion (HSIL, AIS and cancer)
ICER: incremental cost‐effectiveness ratio
LR: likelihood ratio
LSIL: low‐grade intraepithelial lesion
MD: medical doctor
NKCx: Swedish National Cervical Screening Registry
NPV: negative predictive value
PPV: positive predictive value
SEK: Swedish krona
TN: true negative
TP: true positive

**Key message**

Liquid‐based sample for co‐testing is a feasible and cost‐effective alternative to colposcopy for follow‐up of histological LSIL, with no missed cancer cases.

## 1 INTRODUCTION

With convincing evidence of more effective cervical cancer screening through human papillomavirus (HPV) testing versus cervical cytology,^1^ HPV‐based screening programs have been implemented around the world. In Sweden, primary screening with HPV analysis has been recommended since 2015, and the national guidelines were last updated in 2025.^2^ Since HPV analysis is both more sensitive^1^ and less specific^3^ compared with cytology, the introduction of HPV‐based screening has resulted in more women being referred for colposcopy examinations.^4^, ^5^ Alongside the increased detection of high‐grade squamous intraepithelial (HSIL) or more severe (HSIL+) lesions, including HSIL, adenocarcinoma in situ (AIS), and cancer, the number of detected low‐grade squamous intraepithelial lesions (LSILs) has risen.^5^ Consequently, the number of women requiring treatment and additional clinical follow‐up has escalated. Since the implementation of HPV‐based screening, colposcopy units have struggled to follow the guidelines, as more women require investigation, treatment, and follow‐up. It is of utmost importance that all women with aberrant screening results obtain colposcopy in time and that women with HSIL+ receive timely treatment. The follow‐up of histological LSIL is of lower priority but still necessary.

A colposcopy within 12–24 months (including a biopsy for histology in case of an aberrant examination), together with a liquid‐based sample for HPV analysis and cytology (co‐testing), is recommended as follows: follow‐up to histological LSIL in Sweden. This approach is primarily based on professional experience, since evidence on the optimal follow‐up for LSIL is lacking. Even so, most LSILs are due to self‐healing HPV infections, progress slowly, and seldom lead to cancer.^6^ Studies have reported LSIL regression rates as high as 88.5%.^7^ Eventual LSIL progression in follow‐up is associated with HPV persistence.^8^ HPV‐testing has also been shown to have a high negative predictive value (NPV) for HSIL+ in cervical screening.^9^

This study aimed to investigate whether it is safe to replace colposcopy with co‐testing or HPV‐alone testing as the primary follow‐up method for histologically confirmed LSIL. The primary outcome was to estimate the diagnostic performance of the strategies of co‐testing (HPV + cytology) and HPV‐alone in detecting histological HSIL+ in the follow‐up to histological LSIL. The secondary outcome was to perform a cost‐effectiveness analysis of these strategies.

## 2 MATERIAL AND METHODS

### 2.1 Study design, data sources, and study population

This is a retrospective cohort study on follow‐up data of women with histological LSIL. The study population was identified through the Swedish National Cervical Screening Registry (NKCx),^10^ which covers all data on invitations, cervical cytology results, HPV results, and cervical histological results from all Swedish laboratories. All women of screening age (23–70 years) between January 1, 2017, and December 31, 2018, who had a histological LSIL with no previous or concomitant histological HSIL+ at the time of LSIL diagnosis were identified. Follow‐up data until December 28, 2023, were retrieved. Women with initial histological LSIL and histological diagnosis at follow‐up within 9 months and/or after 30 months were excluded to best analyze the cases with follow‐up in line with recommendations at the time of the study.^2^

Women with results from all three test methods (HPV, cytology, and histology) at follow‐up were identified. The results at the first follow‐up visit were analyzed, and the most aberrant histology was used as the outcome measure. Only HPV and cytology results within weeks after colposcopy at follow‐up were included in the analysis to identify cases managed according to guidelines. The strategies compared are co‐testing (HPV + cytology analyzed and regarded as positive if either test is positive), HPV‐alone (only HPV analyzed), and colposcopy (co‐testing and biopsy).

Further analysis was performed assuming that women without histological results from colposcopy had normal examinations and were thus considered to have normal histology at follow‐up.

### 2.2 Statistical analysis

Cumulative incidence was calculated for HSIL+, HSIL, AIS, and cancer as the proportion of individuals who developed disease during the study period. To estimate the diagnostic performance of co‐testing and HPV‐alone in detecting histological HSIL+, the sensitivity, specificity, positive predictive value (PPV), negative predictive value (NPV), positive (LR+), and negative (LR−) likelihood ratios with 95% exact binomial confidence intervals (95% CI) were calculated. Approximate area under the curve (AUC) with 95% CI was also calculated.^11^

The diagnostic performance of co‐testing was compared with that of HPV‐alone in detecting HSIL+. The McNemar test was used to compare sensitivity, specificity, LR+, and LR− with 95% CI for the difference. The Wald test with a generalized estimating equation to account for within‐subject correlation was applied to compare PPV and NPV with 95% CI for the difference. The difference in the AUCs was estimated as follows:

*AUC*_difference_ = *AUC*_co‐testing_ – *AUC*_HPV‐alone_ with a 95% CI. A two‐sided *p* < 0.05 was considered significant. Statistical analyses were performed using R (version 4.3.3).

### 2.3 Estimation of costs and modeling cost‐effectiveness

Cost‐effectiveness was modeled from a healthcare perspective for the 4213 women with results from all three tests, as well as for the 9315 women (In the model, costs are estimated based on 9450 women in order not to bias cost‐effectiveness results by comparing groups of different sizes. This is motivated by the assumption that the 135 women missing data had examinations with normal histological results.) with co‐testing as follow‐up. Costs and effects were modeled for each of the three strategies (co‐testing, HPV‐alone, and colposcopy) and then compared in terms of incremental cost‐effectiveness. To estimate cost‐effectiveness, healthcare resource use resulting from the included strategies was measured and valued. For co‐testing and HPV‐alone, this included sampling by a midwife followed by laboratory analyses. Those with positive results were referred to a gynecologist for colposcopy, followed by histological analysis. In the third strategy, colposcopy including co‐testing was performed by a gynecologist, followed by laboratory analyses including histology.

Hence, the relevant resource use items identified were the cost for midwife visits for sampling, the cost for performing colposcopies, and the costs for analyzing HPV, cytology, and histology samples. Costs occurring outside healthcare, such as work absence or health effects following colposcopy, were not included.

Costs for midwife visits were estimated based on data on wages (including social fees) for midwives collected from administrative sources for Region Örebro County and the assumed time required for sampling. Colposcopy costs were retrieved from the regional *Cost per Patient* database. Laboratory costs were estimated by the Laboratory Medicine Clinic at Örebro University Hospital. Table 3 presents unit costs. Costs were initially measured in SEK at the 2023 price level and then converted to EUR at an exchange rate of 1 EUR = 11.48 SEK, corresponding to the average 2023 exchange rate.^12^

Effects are measured by the number of HSIL+ respectively detected by the three strategies. Cost‐effectiveness is expressed by the incremental cost‐effectiveness ratio (ICER), which is, the cost of achieving one more unit of the effect. The ICER here corresponds to the cost of detecting one more HSIL+. When comparing more than two mutually exclusive options, strategies are ordered by increasing effectiveness and then compared pairwise.^13^ Hence, each ICER expresses the extra cost of one additional unit of the effect compared to the next‐most effective strategy.

The ICERs estimated here correspond to Equations (1) and (2), where *A* represents the strategy detecting the least, *B* the next‐least, and *C* the highest number of HSILs+.

(1) $$\frac{\left({\text{Cost}}^{B}-{\text{Cost}}^{A}\right)}{\left(\#\text{HSIL}{+}^{B}-\#\text{HSIL}{+}^{A}\right)}$$

(2) $$\frac{\left({\text{Cost}}^{C}-{\text{Cost}}^{B}\right)}{\left(\#\text{HSIL}{+}^{C}-\#\text{HSIL}{+}^{B}\right)}$$

## 3 RESULTS

### 3.1 Data on women with results from all three test methods

**FIGURE 1:**
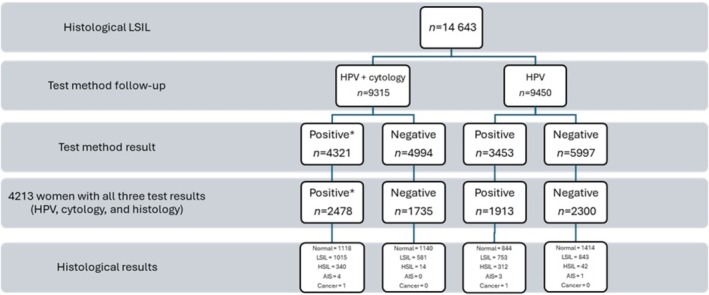
Follow‐up tests for the 14 643 women with histologically confirmed LSIL. (*The HPV test and/or cytology test show positive findings.)

In total, 14 643 women with histological LSIL in cervical biopsy were included in the study. Among these women, 6398 had histological results from colposcopy at follow‐up; of these, 5934 women had cytology results and 4347 women had HPV test results. Ultimately, 4213 women had results in all three analyses: HPV test, cytology, and histology. Follow‐up histology among these 4213 women was distributed as follows: 53.6% (2258 women) exhibited normal findings, 37.9% (1596 women) showed LSIL, and 8.4% (354 women) showed HSIL. Additionally, four women were diagnosed with AIS and one was diagnosed with cancer. In total, 8.5% of the women were diagnosed with HSIL+ in follow‐up (Figure 1).

If a co‐testing strategy had been employed, with colposcopy conducted only for positive findings from either or both tests, 2478 colposcopies would have been required, yielding an HSIL+ incidence rate of 8.2% (345/4213) cases, a sensitivity to detect HSIL+ of 96% (345/359), and a specificity of 45% (Table 1). In an HPV‐alone strategy, 1913 colposcopies would have been performed, with an HSIL+ incidence rate of 7.5% (316/4213), a sensitivity to detect HSIL+ of 88% (316/359), and a specificity of 59%. The sensitivity of HPV‐alone compared with co‐testing was significantly lower (*p* < 0.0001), while the specificity was significantly higher (*p* < 0.0001) (Table 2). For co‐testing, the PPV was 0.14 (95% CI: 0.13–0.15) and the NPV was 0.99 (95% CI: 0.98–0.99). For HPV alone, compared with co‐testing, the PPV was significantly higher, at 0.17 (95% CI: 0.15–0.18; *p* < 0.0001), and the NPV was significantly lower, at 0.98 (95% CI: 0.97–0.99; *p* < 0.0001) (Table 2).

**TABLE 1:** Histological outcomes in 4213 women with LSIL in relation to co‐testing with HPV and cytology or HPV alone at 1 year follow‐up.

Category	HPV + cytology		HPV	
	Positive, *n* (%) (*n* = 2478)	Negative, *n* (%) (*n* = 1735)	Positive, *n* (%) (*n* = 1913)	Negative, *n* (%) (*n* = 2300)
Normal	1118 (45.10)	1140 (65.70)	844 (44.10)	1414 (61.50)
LSIL	1015 (41.00)	581 (33.50)	753 (39.40)	843 (36.70)
HSIL	340 (13.70)	14 (0.81)	312 (16.30)	42 (1.83)
AIS	4 (0.16)	0 (0.00)	3 (0.16)	1 (0.04)
Cancer	1 (0.04)	0 (0.00)	1 (0.05)	0 (0.00)

Abbreviations: AIS, adenocarcinoma in situ; HPV, human papilloma virus;, LSIL, low grade intraepithelial lesions; HSIL, high grade intraepithelial lesions.

**TABLE 2:** Diagnostic performance of the strategies of co‐testing and human papillomavirus testing alone versus colposcopy in detecting histological high‐grade squamous intraepithelial lesions+ in follow‐up after histological low‐grade squamous intraepithelial lesion detection.

	Co‐testing (*n*)	HPV‐alone (*n*)
*Diagnostic performance measures*		
TP	345	316
FP	2133	1597
TN	1721	2257
FN	14	43

	Co‐testing (95% CI)	HPV‐alone (95% CI)	*p* value of difference between performance
Sensitivity	0.96 (0.94, 0.98)	0.88 (0.85, 0.91)	<0.0001
Specificity	0.45 (0.43, 0.46)	0.59 (0.57, 0.60)	<0.0001
PPV	0.14 (0.13, 0.15)	0.17 (0.15, 0.18)	<0.0001
NPV	0.99 (0.99, 0.10)	0.98 (0.98, 0.99)	<0.0001
Approximate AUC	0.704 (0.690, 0.717)	0.733 (0.714, 0.751)	<0.05

Abbreviations: AUC, area under the curve; FN, false negative; FP, false positive; HPV, human papillomavirus testing; HSIL+, high‐grade squamous intraepithelial lesion; NPV, negative predictive value; PPV, positive predictive value; TN, true negative; TP, true positive.

Performing colposcopy as the primary follow‐up strategy for histological LSIL result in an additional 14 cases of HSIL+ (3.9% of all HSILs+) compared to co‐testing and 43 additional cases of HSIL+ (12.0% of all HSILs+) compared to the HPV‐alone strategy (Figure 1; Table 2). However, this approach required 1735 out of 2487 (69.8%) more colposcopies compared with what the co‐testing strategy would have required, or 2300 out of 1913 (120.2%) more than the HPV‐alone strategy.

The LR+ of co‐testing was 1.74 (95% CI: 1.68–1.80) and the LR− was 0.09 (95% CI: 0.05–0.15). The LR+ of HPV‐alone was significantly higher than that of co‐testing, at 2.12 (95% CI: 2.01–2.24; *p* < 0.0001), and the LR− was significantly lower, at 0.20 (95% CI: 0.15–0.18; *p* < 0.0001). The approximate AUC for co‐testing was 0.7038 (95% CI: 0.6902–0.7168), significantly lower (*p* < 0.05) than that of HPV‐alone, at 0.7329 (95% CI: 0.7137–0.7513) (Table 2).

### 3.2 Data on all follow‐up for women with co‐testing and HPV‐alone but without histological follow‐up

Analyzing the data assuming that women without histological results from colposcopy had normal examinations, that a biopsy was performed, and that the histology would also have been normal, the incidence rate of HSIL+ was 3.85% (359/9315) for co‐testing. In contrast, when performing HPV‐alone, the incidence rate of HSIL+ was 3.7% (359/9450). Using a co‐testing strategy, the LSIL regression rate was calculated to be 79.0% (7360/9315), while 17.1% of women had persisting histological LSIL. In comparison, an HPV‐alone strategy would have resulted in an LSIL regression rate of 79.3% (7495/9450) (Figure 1), while 16.9% would have had persisting histological LSIL.

### 3.3 Cost‐effectiveness

Table 3 presents estimations of costs per strategy. The upper part shows estimations for women with results from all three strategies, while the lower part presents estimations including women with no histological results, assuming that absence of results indicates normal histology and that all women had taken all tests.

**TABLE 3:** Unit costs and cost estimations for the three strategies of HPV‐alone, co‐testing, and co‐testing + colposcopy in detecting high‐grade squamous intraepithelial lesions, or more, HSIL+ in women aged 23–70 diagnosed with histological low‐grade squamous intraepithelial lesion.

Strategy resource item	Unit cost (€)	Frequency	Total cost	Cost per woman (€)	# of HSILs+ detected	Cost per HSIL+ detected
*(A) Women with results from all three test methods*						
**HPV‐alone**			1 433 845	340	316	4537
Midwife visit	9.93	4213	41 836			
HPV analysis	19.16	4213	80 737			
Colposcopy (MD)	608.80	1913	1 164 630			
Biopsy, histology	76.66	1913	146 641			
**Co‐testing**			1 979 664	469	345	5738
Midwife visit	9.93	4213	41 836			
HPV analysis	19.16	4213	80 737			
Cytology analysis	37.63	4213	158 538			
Colposcopy (MD)	608.80	2478	1 508 601			
Biopsy, histology	76.66	2478	189 951			
**Co‐testing + colposcopy**			3 127 088	742	359	8710
HPV analysis	19.16	4213	80 737			
Cytology analysis	37.63	4213	158 538			
Colposcopy (MD)	608.80	4213	2 564 866			
Biopsy, histology	76.66	4213	322 948			
*(B) Including women with no histological results*						
**HPV‐alone**			2 641 808	280	316	8360
Midwife visit	9.93	9450	93 841			
HPV analysis	19.16	9450	181 098			
Colposcopy (MD)	608.80	3453	2 102 179			
Biopsy, histology	76.66	3453	264 690			
**Co‐testing**			3 592 391	380	345	10 412
Midwife visit	9.93	9450	93 841			
HPV analysis	19.16	9450	181 098			
Cytology analysis	37.63	9450	355 610			
Colposcopy (MD)	608.80	4321	2 630 616			
Biopsy, histology	76.66	4321	331 226			
**Co‐testing + colposcopy**			7 014 238	742	359	19 538
HPV analysis	19.16	9450	181 098			
Cytology analysis	37.63	9450	355 610			
Colposcopy (MD)	608.80	9450	5 753 140			
Biopsy, histology	76.66	9450	724 390			

Increasing effectiveness in terms of the number of HSILs+ detected is accompanied by increasing costs for follow‐up. To estimate cost‐effectiveness, strategies are compared stepwise by increasing effectiveness. First, the strategy of detecting the next‐least number of HSILs+—that is, co‐testing—is compared with the strategy of detecting the least number of HSILs+—that is, HPV‐alone. For women with complete results, the ICER is estimated as shown in Equation (3); when including women with no histological results, the ICER is estimated as in Equation (4).

(3) $$\begin{array}{c} \frac{\left(\text{€}1\;979\;664-\text{€}1\;433\;845\right)}{\left(345-316\right)}=18\;821\;\mathrm{EUR}\;\mathrm{per}\;\text{additional HSIL}\\ \;+\;\text{detected} \end{array}$$

(4) $$\begin{array}{c} \;\frac{\left(\text{€}3\;592\;391-\text{€}2\;641\;808\right)}{\left(345-316\right)}=32\;779\;\mathrm{EUR}\;\mathrm{per}\;\text{additional HSIL}\\ \;+\;\text{detected} \end{array}$$

Next, the strategy that detected the highest number of HSILs+—that is, co‐testing and colposcopy—is compared with the strategy that detected the next‐highest number of HSILs+—that is, co‐testing.

For women with complete results, the ICER is estimated as shown in Equation (5); when including women with no histological results, the ICER is estimated as in Equation (6).

(5) $$\begin{array}{c} \frac{\left(\text{€}3\;127\;088-\text{€}1\;979\;664\right)}{\left(359-345\right)}=81\;959\;\mathrm{EUR}\;\mathrm{per}\;\text{additional HSIL}\\ \;+\;\text{detected} \end{array}$$

(6) $$\begin{array}{c} \frac{\left(\text{€}7\;014\;238-\text{€}3\;592\;391\right)}{\left(359-345\right)}=244\;417\;\mathrm{EUR}\;\mathrm{per}\;\text{additional HSIL}\\ \;+\;\text{detected} \end{array}$$

## 4 DISCUSSION

This study on different follow‐up strategies for histological LSIL demonstrates that the incidence of HSIL+ is 8.5% in primary colposcopy, 8.2% in co‐testing, and 8.2% in the HPV‐alone strategy. The co‐testing and HPV‐alone strategies would have required 41.2% and 54.6%, fewer colposcopies, respectively, compared to the current strategy, which includes a primary colposcopy visit in all cases with histological LSIL. Applying the co‐testing strategy, 3.9% of HSIL+ cases would have been missed; applying the HPV‐alone strategy, 12.0% of HSIL+ cases would have been missed. No cancer cases would have been missed by using the co‐testing or HPV‐alone strategy for follow‐up. The study design did not allow a review of the histological diagnosis on HPV‐negative cases, although this might have been of interest.

Increasing effectiveness comes at an increasing cost. The addition of colposcopy to co‐testing for all women—compared with colposcopy only among women positive for HPV or cytology tests—brought a substantial increase of 81 959 EUR per additional HSIL+ detected. As there is no cost‐effectiveness threshold for the detection of one additional HSIL+, it is impossible to directly draw firm conclusions on the cost‐effectiveness. Instead, the ICER must be interpreted in terms of the decision‐maker's willingness to pay. Importantly, no cancer cases would have been missed if the co‐testing or HPV‐alone strategies had been used, with colposcopy only for women with positive test results. It should be noted that colposcopies without clinical relevance bring additional costs in terms of health opportunity costs—not captured in the cost‐effectiveness model—if these procedures delay treatment of other patients, as real‐world clinical capacity is limited.

Excisional treatment of LSIL has been replaced with active surveillance with regular colposcopy examinations, since LSILs very seldom progress to HSIL+. A 2021 systematic review found that approximately 60% of LSIL lesions regress spontaneously, while 11% progress to higher‐grade lesions. The progression rate from LSIL to CIN3+ was only 2%, and the incidence of cervical cancer was as low as 0.03%. Most studies included in the review had follow‐up periods of 24 months.^14^ These results are consistent with our data on all confirmed histological LSIL cases, which demonstrate an incidence of 3.85% for HSIL+ (including CIN2) in follow‐up, with no cases of invasive cancer observed. Our study also observed a very high LSIL regression rate (almost 80%), further supporting the notion that routine colposcopy may be unnecessary. Moreover, many women experience significant anxiety when referred for colposcopy,^15^ underscoring the need to avoid overuse of this clinical procedure.

Sensitivity and NPV were higher with co‐testing, but specificity and PPV were higher with HPV‐alone. The AUC confirmed that HPV‐alone better differentiated between high‐grade and low‐grade lesions, which is the main purpose of follow‐up. It should also be noted that the major reason for screening and follow‐up is efficient prophylaxis of cancer. Missing some HSIL cases (without cancer) might be acceptable for a follow‐up strategy that is easy, affordable, and available for all cases.

HPV‐negative HSIL is associated with a lower risk of cancer and higher likelihood of regression.^14^, ^16^, ^17^, ^18^, ^19^ A negative HPV‐test might precede HSIL regression, and the diagnosis of CIN2 is known to be a heterogeneous diagnosis with great interobserver variability and poor reproducibility.^20^, ^21^ A large retrospective routine clinical follow‐up study on HPV‐negative women with HSIL cytology (Pap smear) showed a prevalence of 2.7% (15/533). HPV‐negative histological CIN2+ (including one case of squamocolumnar cancer) and concluded that the results confirmed the high sensitivity of HPV testing in diagnosing histological CIN2+.^22^ In our study, 1.9% (43/2300) of histological HSILs+ were HPV‐negative, which aligns with the results of the aforementioned study. Possible explanations for HPV‐negative HSIL+ might include inadequate sampling, lesion regression, low viral loads, and HPV infections with genotypes not included in the test panel.^23^ These findings might be interpreted to diminish the importance of missing a small number of HPV‐negative HSIL+ cases.^17^

A major strength of this study is that it includes all samples from all laboratories in Sweden for women diagnosed with histological LSIL, along with their follow‐up samples, providing a uniquely comprehensive dataset. One limitation is that not all women were treated in line with clinical guidelines, reflecting real‐world data. While this could be seen as a weakness when trying to assess guideline effectiveness under ideal conditions, it is a strength for understanding how guidelines are applied in practice. Furthermore, although the registers are robust, they do not allow us to confirm with certainty that all women underwent follow‐up and colposcopy if no biopsy was taken. Also, the study is based on a national register and is connected to medical records where procedures are documented and uses a retrospective design. However, as results are in line with the national guidelines in other countries, selection bias is unlikely.^24^, ^25^, ^26^

This register‐based study indicates that not all women were followed up according to guidelines only about two‐thirds had results from cytology or HPV tests, and less than one‐third received histology results from punch biopsies during colposcopy. However, punch biopsies were not mandatory at that time, according to the guidelines, when the colposcopy appearance was normal. These findings might also reflect the study design, as all potentially relevant data is not available in registers.

## 5 CONCLUSION

Primary follow‐up of histological LSIL by colposcopy can be substituted with liquid‐based samples for co‐testing, which appears to be a feasible strategy with no missed cancer cases. The cost‐effectiveness analysis supported this conclusion, as the incremental cost for detecting one additional HSIL+ is not considered justifiable. Based on our findings, even HPV‐alone testing may be sufficient and transitioning to HPV self‐sampling should be studied as a follow‐up strategy. Such an approach may further increase cost‐effectiveness by reducing the need for clinical visits and procedures.

## AUTHOR CONTRIBUTIONS

Riina Aarnio and Lovisa Bergengren conceptualized the study and determined the methodology. Linda Ryen and Vasudha Ahuja were responsible for the formal analysis and data curation. All authors together validated the results. All authors contributed to writing, editing, and critically revising the manuscript, as well as preparing of visualizations. Lovisa Bergengren oversaw project administration, supervised the project, and secured funding for the study.

## FUNDING INFORMATION

This work received support from the Region Örebro County Research Committee OLL‐1014240, OLL‐1004308.

## CONFLICT OF INTEREST STATEMENT

All the authors have no conflicts of interest.

## ETHICS STATEMENT

Ethical approval was given by the Swedish Ethical Review Board (No. 2023‐05433‐01) 10 of October 2023. All data were extracted retrospectively and anonymized/unidentified. The study did not influence the follow‐up and potential care, and individual informed consent was not required.

## Data Availability

The data that support the findings of this study are available on request from the corresponding author. The data are not publicly available due to privacy or ethical restrictions.
